# Are infant feeding practices correlates of nutritional status of 6-23 months old babies in a rural community in Nigeria?

**DOI:** 10.4314/ahs.v25i4.24

**Published:** 2025-12

**Authors:** Tope Joseph Olukayode, Motunrayo Funke Olumakaiye, Caleb Aderemi Adegbenro

**Affiliations:** 1 Department of Community Health, Faculty of Clinical Sciences, Obafemi Awolowo University, Ile-Ife, Nigeria; 2 Department of Human Nutrition and Dietetics, Faculty of Basic Medical Sciences, Obafemi Awolowo University, Ile-Ife, Nigeria; 3 Public Health Programme, Faculty of Basic Medical and Health Sciences, Bowen University Iwo, Osun State, Nigeria

**Keywords:** Exclusive Breast Feeding, Minimum Meal Frequency, Minimum Dietary Diversity, Stunting, Wasting, Underweight

## Abstract

**Background:**

Infant Feeding Practices (IFPs) and Nutritional Status (NS) of children between 6 – 23 months have been a matter of concern, and these were investigated in a rural community in Nigeria.

**Methods:**

Data were collected from 368 mothers with children 6–23 months. IFPs and anthropometry were investigated among the children. Wasting (WHZ), stunting (HAZ), and underweight (WAZ); Exclusive Breastfeeding (EBF), Minimum Meal Frequency (MMF), and Mean Dietary Diversity (MDD) were determined. Data were analyzed using descriptive statistics and logistic regression, and significance was set at p<0.05.

**Results:**

Wasting was 22.9%, 28.3% stunted, and 17.1% underweight. Children without EBF were thrice more likely to be wasted (OR = 3.454, 95%CI = 1.667 - 7.156), twice more likely to be stunted and underweight (OR = 2.189, 95%CI = 0.975 - 4.918). Those with low MMF were thrice more likely to be wasted (OR = 3.451, 95%CI = 1.798 - 6.622) and twice more likely to be stunted (OR = 1.674, 95%CI = 0.868 - 3.227). Those with low MDD were thrice more likely to be stunted (OR = 3.295, 95%CI = 0.856 – 12.683) and underweight (OR = 3.295, 95%CI = 0.856 - 12.683). Caregivers with unskilled occupations, who earn lower income, are younger in age, with a lower education level are associated with a higher likelihood of having children with wasting, stunting and underweight.

**Conclusion:**

EBF, MMF, and MDD affected the NS of children 6-23 months in the study area.

## Introduction

Malnutrition in children is a serious global health concern that occurs when a child does not receive adequate nutrition, either in terms of quantity or quality, to support their growth and development^1^. It encompasses both undernutrition and overnutrition, each with distinct consequences for a child's health. Undernutrition in children comprises wasting, stunting and underweight. Wasting is characterized by a rapid loss of body weight, often due to acute malnutrition. It results in a thin and emaciated appearance, indicating a severe nutritional deficit. Stunting occurs when a child experiences chronic malnutrition, leading to impaired growth and development. Stunted children are shorter than their peers and may face cognitive and developmental delays. Underweight on the other hand is a general measure of a child's weight in relation to their age, reflecting both acute and chronic malnutrition^1^. United Nations Children's Emergency Fund (UNICEF) Report^2^ suggested poverty and childcare practices such as feeding practices and responsive caregiving are major factors contributing to malnutrition^3^. Globally, in 2018, stunting affected an estimated 21.9% of children under the age of 5 years, while wasting affected 7.3%. Around 45% of deaths among children under the age of 5 years are linked to undernutrition. These mostly occur in low and middle income countries^4^. According to the World Health Organization (WHO), UNICEF and World Bank joint child malnutrition estimates report^5^, a high prevalence level (36%) of stunting of children under five years was reported in Africa and this remains a public health problem^6^.

In Nigeria, data from the NDHS^6^ indicated that the country's malnutrition indicators among children under 5 years of age improved over the last decade between 2013 and 2016^7^,^8^. Despite the improvement, the rates are still higher than global rates^4^. However, the indicators suffered a setback afterwards. The extent of wasting reduced from 18% in 2013 to 9% in 2016 and further reduced to 7% in 2018 [USAID 2020]. Unfortunately, it increased to 11.6% in 2023 [UNICEF 2023]. The proportion of children who were stunted declined from 41% in 2008 to 37% in 2013, 32% in 2016^9^,^10^, increased to 37% in 2018 and stabilized afterwards at 32% in 2020 [World Bank, 2020] and 33% in 2023 [UNICEF 2022, NFCMS 2022]. This makes Nigeria adjudged the second-highest burden of stunted children in the world [USAID, 2020, UNICEF, 2023]. Likewise, underweight reduced from 29% in 2013 to 21% in 2018^6^,^10^ but increased to 25.3% in 2023 [USAID, 2020, UNICEF 2023]. It was also reported that an estimated 2 million children suffer from severe acute malnutrition [NPC-ICF 2019, USAID, 2020, UNICEF 2023]. This decline and lack of progress in reducing undernutrition are exacerbated by armed conflicts, insurgency, insecurity and displacement, which continue to impact a large number of children, particularly in various regions of the north of the country [USAID 2020]. Malnutrition is a direct or underlying cause of 45% of all deaths of under-five children in Nigeria, with children at greater risk of dying from common infections, increasing the frequency and severity of such infections and contributing to delayed recovery. Each year, about one million Nigerian children die before their fifth birthday with malnutrition, a contributing factor of nearly half of these deaths. Although undernutrition has declined, achieving Sustainable Development Goals (SDGs) and Vision 2030 goals of a 40% reduction in the number of children under five who are stunted and a reduction of childhood wasting to less than 5% may still not be reachable unless more is done^11^,^9^,^10^.

Feeding practices have been identified as a contributory factor to undernutrition among under-five children^2^. Interaction between feeding practices and undernutrition can create a potentially lethal cycle of worsening illness and deteriorating nutritional status. Poor nutrition in the first 1,000 days of a child's life leads to stunted growth, which is irreversible and associated with impaired cognitive ability and reduced school and work performance^1^. In Nigeria, 17% of children less than age 6 months are exclusively breastfed. Only 10% of children aged 6-23 months are fed appropriately based on recommended infant and young child feeding (IYCF) practices^6^. Despite many programmes focusing on providing supplementary feeding in the form of specialized food products such as Ready-to-Use Therapeutic Foods (RUTF)^12^,^13^,^14^ and nutritional supplements such as vitamin A supplementation^6^,^15^,^16^ to provide additional nutrients for malnourished children under five years of age, the prevalence of acute and chronic malnutrition in Nigeria has remained a concern^6^. Ondo State is one of the states in Nigeria and the report of the 2013 Nigeria Demographic and Health Survey^17^ showed that the state had one of the worst indices for stunting and underweight in the southwestern geopolitical zone of the country. This spurred the State Government to start a comprehensive nutrition programme tagged “Nutrition Rebirth”, which was implemented between 2015 and 2017. This programme targeted under-five children to reduce the prevalence of undernutrition, which was estimated to be 6.6% for wasting, 24% for stunting and 13.4% for underweight^17^. Some of the activities in the programme included; routine growth monitoring; vitamin supplementation and distribution of zinc sulfate; live educational programme on exclusive breastfeeding, consumption of appopriate diet and water, sanitation and hygiene (WASH) on the State's radio stations (Positive FM 102.5 and Ondo State Radiovision Corporation/OSRC 96.5 FM); zonal training of health workers on infant and young child feeding as well as on International Code of Marketing of Breast Milk Substitutes^18^. Another important component of the Ondo State nutrition programme was the production of blended complementary foods (BCF) sourced from locally available and affordable food materials for the management of moderate acute malnutrition (MAM) cases^18^.

Dietary and non-dietary factors play important roles in determining the nutritional status of children under five, for this study, both were considered. Dietary factors in this context referred to feeding practices which were exclusive breastfeeding^19^,^20^, continued breastfeeding^21^, the introduction of appropriate complementary foods^22^,^23^,^24^, meal frequency^25^,^26^ and dietary diversity^27^,^28^,^29^,^30^,^31^ while the non-dietary factors considered were socio-economic factors^32^,^33^. For this study, the association between caregivers' feeding practices, socioeconomic factors and nutritional status of children between 6 - 23 months were investigated.

## Material and methods

### Study Area

The study was conducted in rural communities at Ose Local Government Area (LGA) of Ondo State, Southwest of Nigeria with headquarters at the town of Ifon. The LGA is made up of communities including Ikaro, Okeluse, Ijagba, Imoru, Arimogija, Elegbeka (Ikaro), Ogberuwen, Ute, Ifon, Omi-alafa, Ugbonla, and Ogbese Falodun to the south; and Ido-ani, Idogun, Ido-isale, Afo, and Imeri to the north. The major activity of this region is farming, mostly based around cocoa and plantain plantation farming^34^. It has an area of 1,465 km^2^ and a population of 144,901 in 2006^35^. The major activity of this region is farming, mostly based around cocoa and plantain plantation farming^34^, with some engaging in caregiving, trading and teaching. The Local government has one general hospital, two private hospitals, four comprehensive basic health centres, nine basic health centres, one private clinic and one health post. The community is a mixture of all the tribes in Nigeria and the common languages spoken were Yoruba and Pidgin English. The major sources of domestic water supply are well water and streams from the Ose River, though there are boreholes scattered across the communities. The main method of waste disposal is dumping hills, which are constantly burnt and toilet facilities are latrines and water closets. The poverty level of the communities is unknown however, Ondo State has the lowest poverty level of 27% in the country^36^. Most women residing in Ose LGA live with their husbands and their children in family houses while few live alone. The major religious groups in the area are Christianity, Islam and Traditional religion.

### Study Design

A descriptive cross-sectional study design using quantitative data was used to determine the influence of caregivers' feeding practices and socioeconomic factors (independent variables) on the nutritional status (dependent variable) of children between 6 - 23 months in villages. The study population comprised primary care-givers (mothers) with children between 6 - 23 months residing in the villages of the LGA. The inclusion criteria were households with children between 6 – 23 months and caregivers who have lived at the selected villages for a minimum of 2 years. While exclusion criteria considered children with disabilities and children whose mothers did not consent to participate in the study.

### Sample Size and Sampling Procedure

The minimum sample size calculated using the formula of Fisher^37^ was 338. With a 9% non-response rate, the sample size was then increased by 30 households to come up with a sample size of approximately 378 households.


$$n=\frac{Z^{2}p(1-p)}{d^{2}}$$

Where,

n = is minimum sample size.
Z =is the table value for standard normal deviate corresponding to 95% significance level (= 1.96).
P = Prevalence of stunted children in Nigeria was, 32.9%^6^
D =Margin error, set at ± 0.05.

Substituting the values in the above formula, the sample size became:


$$\frac{3.84\times 0.329\times 0.67}{0.0025}=338$$

A multistage sampling technique was employed. In the first stage, eight wards were selected using a simple random sampling technique (balloting) from a total of twelve wards in the LGA. In the second stage, there was an average of five villages per ward, out of which, two villages each from the eight wards were selected by balloting, which gave a total of 16 villages. The third stage involved the selection of twenty-three (23) households from each village using a systematic sampling technique with Kth interval once a household had been chosen randomly. In the fourth stage, one eligible woman with a child between 6 - 23 months was selected per household until the desired sample size was achieved. In selected households where there was more than one eligible child, a simple random sampling was used to select the index child.

### Data Collection

Four research assistants who were residents of the LGA were recruited and trained to assist in data collection. The researcher conducted two days of training on data collection techniques relevant to the study on the use of surveys and interview techniques; the procedures of data collection and anthropometric measurements. The field assistants worked in pairs to reduce interpersonal and interview bias. Each administered questionnaire was checked by the researcher to ensure completeness and adequate entry of data. Quantitative data were collected for 2 weeks in April 2017.

### Research Instruments and Measurements

Quantitative data were collected using a pre-tested semi-structured interview schedule, and anthropometric measurements were taken. Minimum Meal Frequency (MMF) was calculated by the proportion of children 6 – 23 months of age who received solid, semi-solid, or soft foods the minimum number of times or more during the previous day as recommended by World Health Organisation^36^. Minimum is defined as 2 times for breastfed infants 6 – 8 months and 3 times for breastfed children 9 – 23 months. Note that “Meals” included both meals and snacks and frequency is based on the caregivers' report. Minimum Dietary Diversity for infant and young child (MDD-IYC) was calculated using the number of food groups consumed by the children. The questionnaire collected information on individual food groups consumed by the children over the past 24 hours as reported by the caregiver/mother. The seven food groups included grains, roots and tubers, legumes and nuts, dairy products (milk, yoghurt, cheese), flesh foods (meat, fish, poultry and liver/organ meats), eggs, vitamin-A-rich fruits and vegetables, and other fruits and vegetables. Children who ate a food group were given a score of 1. Dietary Diversity Score was created by summing the number of individual food groups consumed. A high Individual Dietary Diversity Score (IDDS) was then considered when children consumed four of the seven food groups while a low IDDS was considered when the children consumed less than four food groups over the past 24 hours^38^.

### Nutritional status measurement

The children were weighed naked to the nearest 0.1 kg using a salter and bathroom scale calibrated in 100 g units. Children who were too scared to stand on the scale were weighed together with the mother, and the mother's weight was deducted to obtain the weight of the child. The length of children was measured using a length board; this was done on a firm surface with assistance. These measurements were done to the nearest 0.1 cm. All measurements were taken twice for correctness. WHO child growth chart was used as a reference. The data were categorized according to the standard deviations and Z-scores. The anthropometric indicators; weight for height (WHZ), height-for-age (HAZ) and Weight-for-age (WAZ) were used for wasting, stunting and underweight respectively.

### Data Analysis

The interview schedule was translated into the Yoruba language, which is one of the commonly spoken languages, and later translated back to the English language before analysis. Data were analysed using the SPSS software, version 20. Descriptive and inferential statistics were used where appropriate while binary logistic regression was used to ascertain the predictors of malnutrition and the association between caregivers' feeding practices, socioeconomic factors, and the nutritional status of the children after adjusting for confounding factors. The significance level was recorded at p<0.05.

## Ethical consideration

Approval for the study was obtained from the Health Research and Ethics Committee of the Institute of Public Health, Obafemi Awolowo University, Ile-Ife. Permission was also obtained from the Executive Secretary of Ose Local Government. Verbal informed consent was obtained from respondents with assurance of confidentiality.

## Results

### Socio-demographic characteristics of caregivers of children 6-23months

Out of the 378 data collected, only 368 were included for analysis after the removal of outliers. Table 1 showed the mean age of the caregivers was 24.92± 5.84 years, out of which 20.9% were adolescents aged 10-19 years. Three hundred and twenty-five (88.3%) were married and two hundred and twenty-five (61.1%) were of large families of greater than 6 people living in the household. Furthermore, 85 (23.1%) of them had tertiary education, 51 (13.9%) earned monthly income above ₦ 40000 ($87) and a little above half (204, 55.4%) of the caregivers were skilled workers.

**Table 1:** Socio-demographic characteristics of caregivers of children 6-23months

n = 368		
Variables	n	%
**Age group (years)**		
10 - 19	77	20.9
20 - 29	230	62.5
30 and above	61	16.6
Mean age ± SD	24.92±5.84	
**Ethnicity**		
Hausa	77	20.9
Igbo	61	16.6
Yoruba	84	22.8
Others	146	39.7
**Religion**		
Christianity	89	24.2
Islam	276	75.0
Traditional	3	0.8
**Marital Status**		
Single	42	11.4
Married	325	88.3
Widowed	1	0.3
**Household Size**		
Small (≤3)	13	3.5
Medium (4-6)	130	35.3
Large (>6)	225	61.1
**No of children in the household**		
>3 children	10	2.7
≤3children	358	97.3
**Level of Education**		
No Formal Education	94	25.5
Primary	87	23.6
Secondary	102	27.7
Tertiary	85	23.1
**Monthly Income**		
5000-10000	47	12.8
11000-20000	116	31.5
21000-30000	81	22.0
31000-40000	73	19.8
>40000	51	13.9
**Occupation**		
Unskilled	125	34.0
Skilled	204	55.4
Professional	39	10.6

### Nutritional Status and Infant Feeding Practices of Children 6 – 23 months

**Figure 1:**
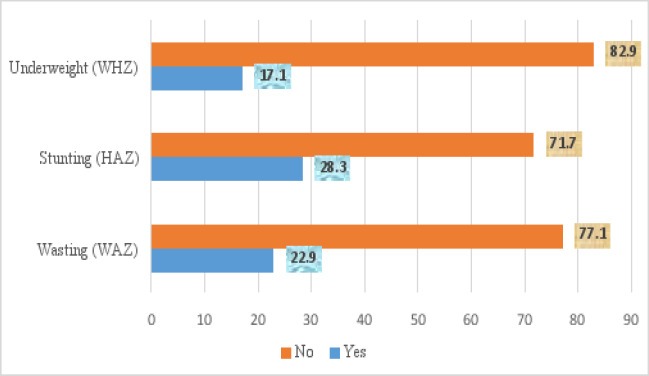
Nutritional Status of Children 6 – 23 months

Nutritional status assessment of the children showed that 84 (22.9%) were wasted, 104 (28.3%) of them were stunted and 64 (17.1%) were underweight (Figure 1). Two hundred and eleven (57.3%) of the caregivers breastfed their children exclusively for 6 months while 320 (87.0%) of the children were fed above the minimum meal frequency (Table 2). The minimum dietary diversity score distribution showed that 306 (83.2%) had dietary diversity scores of less than 4, which was adjudged low.

**Table 2:** Infant Feeding Practices of Children 6 – 23 months

	n=368	
	Frequency (n)	Percentage (%)
**Infant Feeding Practices**		
**Exclusive Breastfeeding (EBF)**		
Yes	211	57.3
No	157	42.7
**Minimum Meal Frequency (MMF)**		
Above	320	87.0
Below	48	13.0
**Minimum Dietary Diversity (MDD)**		
High	62	16.8
Low	306	83.2

### Relationship between Nutritional Status of children and Caregivers' Infant Feeding Practices

In Table 3, a statistically significant proportion of children who were not exclusively breastfed were adjudged wasted (*χ*^2^ = 46.529, p <0.001), stunted (*χ*^2^ = 10.180, p <0.001) and underweight (*χ*^2^ = 22.953, p <0.001) compared to those who were breastfed exclusively. Similarly, those who ate below MMF were wasted (*χ*^2^ = 19.727, p <0.001) and stunted (*χ*2 = 6.532, p= 0.010). Likewise, children with low mean dietary diversity (MDD) were wasted (*χ*^2^ = 7.318, p = 0.004), stunted (*χ*^2^ = 4.069, p = 0.028) and underweight (*χ*^2^ = 5.980, p = 0.008) compared to those rated high. In summary, the results suggest significant associations between infant feeding practices and the nutritional status of 6-23 months old children. Exclusive breastfeeding, meeting minimum meal frequency, and having a high mean dietary diversity are associated with lower prevalence of wasting, stunting, and underweight.

**Table 3:** Chi-square analyses of Nutritional Status of children and Caregivers' Infant Feeding Practices

			n = 368		
Infant Feeding Practices	Yes	No	Total	*χ*^2^/Fishers Exact	P-value
		**Wasting**			
**Exclusively Breastfed (EBF)**					
Yes	21 (10.0)	190 (90.0)	211	46.529	<0.001**
No	63 (40.1)	94 (59.9)	157		
**Minimum Meal Frequency (MMF)**					
Above	61 (19.1)	259 (80.9)	320	19.727	<0.001**
Below	23 (47.9)	25 (52.1)	48		
**Mean Dietary Diversity (MDD)**					
High	6 (9.7)	56 (90.3)	62	7.318	0.004**
Low	78 (25.5)	228 (74.5)	306		
		**Stunting**			
**Exc lusively Breastfed (EBF)**					
Yes	46 (21.8)	165 (78.2)	211	10.180	<0.001**
No	58 (36.9)	99 (63.1)	157		
**Minimum Meal Frequency (MMF)**					
Below	83 (25.9)	237 (74.1)	320	6.532	0.010**
Above	21 (43.8)	27 (56.2)	48		
**Mean Dietary Diversity (MDD)**					
High	11 (17.7)	51 (82.3)	62	4.069	0.028**
Low	93 (30.4)	213 (69.6)	306		
		**Underweight**			
**Exc lusively Breastfed (EBF)**					
Yes	19 (9.0)	192 (91.0)	211	22.953	<0.001**
No	44 (28.0)	113 (72.0)	157		
**Minimum Meal Frequency (MMF)**					
Above	51 (15.9)	269 (84.1)	320		
Below	12 (25.0)	36 (75.0)	48	2.416	0.092
**Minimum Dietary Diversity (MDD)**					
High	4 (6.50)	58 (93.5)	62	5.980	0.008**
Low	59 (19.3)	247 (80.7)	306		

** Significant @ p<0.05

The logistic regression analysis predicting the influence of infant feeding practices on nutritional status indicated that children who were not exclusively breastfed were 3 times more likely to be wasted (OR = 3.454, 95%CI = 1.667-7.156, p = 0.001), twice more likely to be stunted and underweight (OR = 2.189, 95%CI = 0.975- 4.918) compared to those who were exclusively breastfed. Furthermore, those children who ate below MMF were 3 times more likely to be wasted (OR = 3.451, 95%CI = 1.798-6.622, p<0.001) than those who ate above MMF. Similarly, children with low MDD were 3 times more likely to be underweight (OR = 3.295, 95%CI = 0.856-12.683), though not statistically significant at p <0.05 (Table 4).

**Table 4:** Logistic regression analysis of the influence of infant feeding practices on nutritional Status

Feeding Practices	Odd Ratios	Confidence Interval	p-value
	**Wasting**		
**Exclusive breastfeeding (EBF)**			
Yes (RC)	1.000		
No	3.454	1.667 -7.156	0.001**
**Minimum Meal Frequency (MMF)**			
High (RC)	1.000		
Low	3.451	1.798-6.622	<0.001**
**Mean Dietary Diversity (MDD)**			
High (RC)	1.000		
Low	1.379	0.424-4.483	0.593
	**Stunting**		
**Exclusive breastfeeding**			
Yes (RC)	1.000		
No	2.189	0.975 - 4.918	0.058
**Minimum Meal Frequency**			
Above (RC)	1.000		
Below	1.674	0.868-3.227	0.124
**Mean Dietary Diversity (MDD)**			
High (RC)	1.000		
Low	3.295	0.856-12.683	0.082
	**Underweight**		
**Exclusive breastfeeding**			
Yes (RC)	1.000	-	
No	2.189	0.975- 4.918	0.058
**Minimum Meal Frequency**			
High (RC)	1.000	-	-
Low	-	-	-
**Mean Dietary Diversity (MDD)**			
High (RC)	1.000	-	-
Low	3.295	0.856-12.683	0.083

** Significant @ p<0.05

Children of caregivers below age 20 were thrice more likely to be stunted (OR = 3.026, p = 0.001) and 4 times more likely to be underweight (OR = 3.501, p = 0.001). While children of caregivers of Hausa ethnicity were 12 times more likely to be stunted (OR = 11.667, p<0.001) and 21 times more likely to be underweight (OR = 21.356, p <0.001). Similarly, those with no education were 15 times more likely to be stunted (OR = 15.368, p<0.001) and had a high likelihood of being underweight (OR = 20.806, p<0.001). Those whose caregivers were unskilled workers were thrice more likely to be wasted (OR= 2.511, p = 0.011), 13 times the likelihood of being stunted (OR = 12.750, p = 0.001) and 4 times the likelihood of being underweight (OR = 4.097, p = 0.029). Children whose caregivers earn ₦ 20000 and below were 8 times more likely to be stunted (OR = 7.718, p<0.001) and 6 times more likely to be underweight (OR = 5.953, p<0.001). Furthermore, children whose caregivers were traditional worshipers were twice likely to be wasted (OR = 1.658, p<0.001) (Table 5). In summary, considering the odds ratio of logistic regression analyses examining the relationship between caregivers' variables and the nutritional status of 6-23 months old children, caregivers who practised traditional religion, unskilled occupation, earn lower income, younger in age, with lower education level and of Hausa ethnic group are associated with a higher likelihood of having children with wasting, stunting and underweight.

**Table 5:** Logistics Regression analyses showing the relationship between Caregivers' variables and nutritional status of 6-23months old children

Variables	Odds Ratio		Confidence Interval		p-value
			Lower limit	Upper limit	
		**Wasting**			
**Caregivers age group**					
<20 years	0.940		0.536	1.649	0.829
≥20 years (RC)	1.000		--	--	--
**Religion**					
Christianity (RC)	1.000		--	--	--
Islam	0.955		0.564	1.616	0.863
Traditionalist	1.658		0.580	3.689	<0.001**
**Level of Education**					
Not educated	1.267		0.761	2.108	0.362
At least pry education (RC)	1.000		--	--	--
**Occupation**					
Unskilled	2.511		1.234	5.112	0.011**
Skilled (RC)	1.474		0.896	2.425	0.127
Professional	1.000		--	--	--
**Income category**					
<₦20000	1.111		0.704	1.752	0.652
≥₦20000 (RC)	1.000		--	--	--
		**Stunting**			
**Caregivers age group**					
<20 years	3.026		1.756	5.214	<0.001**
≥20 years (RC)	1.000		--	--	--
**Ethnicity**					
Yoruba	0.573		0.231	1.417	0.228
Hausa	11.667		6.002	22.676	<0.001**
Igbo	0.817		0.326	2.045	0.665
Others (RC)	1.000		--	--	--
**Level of Education**					
Not educated	15.368		8.606	27.442	<0.001**
At least pry education (RC)	1.000		--	--	--
**Occupation**					
Unskilled	12.750		2.941	55.281	0.001**
Skilled	3.315		0.760	14.466	0.111
Professional (RC)	1.000		--	--	--
**Income category**					
<20000	7.718		4.294	13.872	<0.001**
≥₦20000 (RC)	1.000		--	--	--
		**Underweight**			
**Caregivers' age group**					
<20 years	3.501		1.665	7.360	<0.001**
≥20 years (RC)	1.000		--	--	--
**Ethnicity**					
Yoruba (RC)	1.000		--	--	--
Hausa	21.356		7.917	57.610	<0.001**
Igbos	1.160		0.316	4.253	0.823
Others	1.397		0.545	3.581	0.754
**Level of Education**					
Not educated	20.806		10.368	41.754	<0.001**
At least primary education (RC)	1.000		--	--	--
**Occupation**					
Unskilled	4.097		1.155	14.540	0.029
Skilled	1.376		0.386	4.901	0.623
Professional (RC)	1.000		--	--	--
**Income category**					
≤₦20000	5.953		2.530	14.010	<0.001**
>₦20000 (RC)	1.000		--	--	--

RC (Reference Category)

** Significant @ p< 0.05

## Discussion

### Nutritional status of children 6 – 23 months old

In Nigeria, insecurity continues to affect the well-being of children through high levels of domestic crime coupled with violent attacks by non-state armed groups in several regions^15^. This could be a possible reason for the lack of progress in reducing undernutrition in the country^14^,^15^. Prevalence of malnutrition regarding wasting, stunting and being underweight has been a persisting problem among children 6 – 23 months in rural areas as indicated in this current study and as reported in different regions in Nigeria^39^,^40^,^41^,^42^,^43^ and other countries^44^,^45^,^46^. This calls for concern despite the “Nutrition Rebirth” comprehensive nutrition intervention programme floated by the state government in the study area from 2015 to 2017^18^ and UNICEF^47^ to mitigate malnutrition among under 5 children and the unacceptable level of undernutrition previously recorded in the state^17^.

From the appraisal of the “Nutrition Rebirth” programme, it was reported that not all the LGAs recorded an improvement in the nutritional status of the children during the intervention period. There was no change in the nutritional status of children in a few LGAs in 2018 compared to 2013^18^, while a few others recorded an increase in the prevalence of malnutrition^18^, which is in tandem with the prevalence of undernutrition in the current study that showed a decline when compared to national values^6^, and an increase when compared with the state value^6^.

The programme utilization and uptake could be investigated. Poor utilization or adoption of similar programmes by mothers, e.g., growth-monitoring practices and exclusive breastfeeding, have been reported in areas of poor socioeconomic development in Africa^18^,^48^. This suggests the need for critical appraisal of the programme to strengthen it in rural areas to achieve its aim.

### Feeding practices and malnutrition

Feeding practices adopted by caregivers are an important aspect of care given to children 6-23 months old. More than half of the caregivers breastfed the babies exclusively for 6 months and an impressive percentage scored above the MMF in complementary feeding, however, the diversity of the diet given to the children was low. Breast milk is an affordable source of food for infants and young children. It is considered the ideal and complete source of nutrition during the first six months of life, tailored to meet the specific needs of a growing baby. Exclusive breastfeeding is advocated for the first 6 months of life for optimal growth. It has been reported that a higher proportion of underweight, stunting, and wasting were found among children who were not exclusively breastfed^49^,^50^. In this current study, children who were not breastfed exclusively were underweight when compared with those who were exclusively breastfed

The World Health Organization^51^ recommends age-specific frequencies of complementary feeding based on estimates of the energy provided by complementary foods. Infants should be fed initially 2-3 times a day between 6-8 months, increasing to 3-4 times daily between 9-11 months and 12-24 months with additional nutritious snacks offered 1-2 times per day, as desired^6^. However, other studies conducted in Nigeria^52^ and India^53^ had earlier reported that mothers did not know the appropriate age-specific frequency of feeding with complementary foods as stipulated by the WHO^51^. Most of them fed their children as frequently as it seemed appropriate to them irrespective of the ages of the infants. This study corroborated the finding that infants who had higher meal frequency were better off in their nutritional status^39^,^54^. A shortfall in meal frequency could lead to malnutrition. Nutritionists have long recognized dietary diversity as a key element of high-quality diets, the higher the dietary diversity score the better the diet quality^30^,^31^ and it is also a proxy for nutrient adequacy of the diet^54^. The low MDD recorded could be attributed to a lack of knowledge of the importance of dietary diversity and probably a lack of production and access to diverse foods in the study area due to plantations around. Low dietary diversity is usually found around plantations and marginalised areas^62^. Less diverse foods may result in inadequate intake of vital nutrients, which is one of the major causes of undernutrition^55^. Malnutrition is experienced by the poorest households due to cereal-based, monotonous diets that lack dietary diversity. Some leading causes for low dietary diversity are decreased dependence on own production, increased purchasing food at markets, lack of suitable lands to cultivate, agro-commercialisation, less knowledge of food and nutrition, low income and high prices of food^62^. The significant independent predictors for wasting were EBF and MMF, while the three IFPs investigated had the likelihood of impacting the wasting, stunting and underweight statuses of the children. The result of this study contradicted the findings in India which recorded no association between wasting and IFPs^56^,^57^.

### Determinants of malnutrition among children 6 – 23 months old

The characteristics of the caregivers investigated showed that mothers' age group, religion, education level, occupation, monthly income and ethnicity were determinants of wasting, stunting and underweight in children 6-23 months old. In the current study, children born of mothers less than 20 years old were more stunted and underweight compared to children whose mothers were 20 years and above. One in 5 of the caregivers was an adolescent in the study area, which is a matter of concern because teenage pregnancy is on the increase^30^,58. In 2021 Nigeria's Adolescent pregnancy was 106 per 1000 and showed an increasing rate^67^. Mothers below age 20 are typically young adolescents. Young caregivers often lack experience and skill in child care and nurturing, which often results in potentially adverse health outcomes for their children. Similarly, adolescent mothers who are malnourished and not fully developed tend to have malnourished children. An underweight woman has a high likelihood of giving birth to a low-weight baby, which may not grow to its full potential. These corroborated studies conducted in Ethiopia^59^,^60^,^61^,^62^, which reported that children by younger women suffer more from undernutrition. Nutrition-sensitive intervention aimed at delaying the age at marriage, and first pregnancy and improving knowledge on reproductive health could help to mitigate teenage pregnancy. Delaying pregnancy may prevent the intergenerational cycle of undernutrition^63^.

Mother's level of education was also a strong factor in determining undernutrition. Low levels of education, especially among women, can contribute to poor nutrition practices. This finding is however at variance with the study conducted in Ethiopia^64^ that the risk of being underweight in children was less likely among children whose mothers have no formal education while it is in tandem with the study in Kenya^65^ and Nigeria^66^,^67^ suggesting that investing in mother's education leads to better and improved nutritional health care for the children. Investing in women by empowering them through education^68^,^69^ would influence the food security of the households, which invariably would influence dietary diversity, and improve the nutritional status of household members including 6-23 months old children.

Caregivers who earn less per month have children who are likely to be undernourished. This highlights the importance of finances in raising the standard of children. This is close to the finding in a study conducted in Sri Lanka^70^, which concluded that mothers with low income were thrice more likely to have underweight children. Occupation and income go hand in hand, the better the occupation (skilled), the higher the income and the better the dietary diversity.

While Ondo state is adjudged to have the lowest rate of poverty^36^, the poverty level of these specific communities investigated is unknown. However, from the study, the parameters (income, level of education and occupation) investigated were low compared to studies from other regions in the country^32^,^33^. This could be attributed to the predominant occupation, which is farming of cocoa and plantain plantations, which are seasonal crops. Lack of financial resources can limit access to nutritious food, healthcare, and sanitation facilities thereby contributing to high rates of undernutrition.

Apart from the nutrition-specific activity of production of blended complementary foods (BCF) sourced from locally available and affordable food materials for the management of moderate acute malnutrition (MAM) cases, there was a live radio educational programme as part of the nutrition-sensitive strategy adopted to enlighten the caregivers/mothers on IYCF practices. The question is, how many of these rural women have radio access? Additional exposure to culturally appropriate messages and outreach programmes may have helped to increase uptake.

## Conclusion

Evidence from this study demonstrated that the underlying determinants of the nutritional status of the index children identified included the mother's age, level of education, occupation, income and ethnicity. The intermediate determinants are various indices of caregiver's feeding practices that contributed to the good nutritional status of children in this study setting included exclusive breastfeeding, meeting minimum meal frequency, and achieving minimum dietary diversity. Promotion of these proper feeding practices will lead to improved child health and food intake, which will in turn promote good nutritional status of children between 6 - 23 months.

Mother's education and skilled occupation came out clearly as significant determinants of malnutrition. It is therefore recommended to target and enable women to participate in educational and skill acquisition programs to reduce malnutrition in young children. Improving the nutrition education and skill empowerment of women whose ages are less than 20 years should be urgently looked into as reported by WHO^71^ that 25% of women have their first child before the age of 19 years and 21% were recorded as caregivers/mothers in this study area, 28% in Bangladesh^55^ and 12% in Indonesia^63^.

Addressing malnutrition in this study area requires both nutrition-sensitive and nutrition-specific interventions. Early identification and intervention are crucial to mitigate the long-term consequences of malnutrition on a child's health and development. Addressing this holistically will go a long way in preventing the vicious cycle of malnutrition.

### Impact of findings on reaching global nutrition targets

The global nutrition community has set specific targets to address malnutrition^1^. These targets include reducing stunting, wasting, and underweight among children under 5, as well as addressing other aspects of nutrition, such as exclusive breastfeeding and IYCF^1^. Malnutrition among children under the age of 5 has significant implications for achieving global nutrition targets and the Sustainable Development Goals (SDGs)^72^. The impact is multifaceted and affects various aspects of well-being.

From the available Nigeria data on child undernutrition^14^, there has not been significant improvement towards achieving the global nutrition targets^1^ and SDG^72^ because the statistics are higher than global targets. However, in the study area, the data are higher in wasting and lower in stunting and underweight compared to national data. This has implications for the overall development of the children. Children in Nigeria have an extremely high risk of exposure to climate and environmental shocks. In 2022, the country experienced the worst flooding in a decade, affecting an estimated 1.9 million children in 34 out of the 36 states^14^. Insecurity continues to affect the well-being of children in Nigeria. An estimated 9.3 million people, including 5.7 million children, are affected by conflicts^14^, which included the region of this study. All these exacerbated the worsening condition of children's health status.

There is an urgency to identify and intensify interventions known to reduce malnutrition if Nigeria is to achieve its national goals for the SDGs. Efforts to address malnutrition among children 6 – 23 months in this study involve a combination of interventions, including improving access to nutritious food, promoting breastfeeding and feeding practices, and addressing socio-economic determinants. Similarly, addressing vulnerability shock is very important. Achieving global nutrition targets requires coordinated location-specific actions involving governments, non-governmental organizations, and the private sector.

### Study's limitations

There is a paucity of previous data from the sampled population. Data collected were based on caregivers' reports which are subject to bias. The project was self-sponsored, hence the limited sample size. The sample size was based on stunting rates, rather than wasting rates, which tend to be lower and may not be a full representation of the population in the communities investigated. Nevertheless, the points discussed represent promising mechanisms for improving infant and young child nutritional status in rural communities.

### Future research directions

There is no disaggregated data by location on the nutritional status of the 6 -23 months old children in the state, likewise, post-intervention data. Having relevant and current data/information on this age group is essential for tracking progress, effectiveness of existing programmes and areas of further intervention in the state.
